# Investigations of changes in the arabinogalactan proteins (AGPs) structure, size and composition during the fruit ripening process

**DOI:** 10.1038/s41598-020-77749-w

**Published:** 2020-11-26

**Authors:** Agata Leszczuk, Adrian Zając, Magdalena Kurzyna-Szklarek, Justyna Cybulska, Artur Zdunek

**Affiliations:** 1 Institute of Agrophysics, Polish Academy of Sciences, Doświadczalna 4, 20-290 Lublin, Poland; 2 Department of Functional Anatomy and Cytobiology, Maria Curie-Skłodowska University, Akademicka 19, 20-400 Lublin, Poland

**Keywords:** Biochemistry, Biological techniques, Molecular biology, Physiology, Plant sciences

## Abstract

Arabinogalactan proteins (AGPs) are ubiquitous cell wall and plasma membrane components and are characterised by extensive glycosylation and heterogeneity of their carbohydrate and protein units. The aim of the study was to evaluate the structural features of AGPs present in apple fruits at different stages of the ripening process. AGPs were extracted using the Yariv reagent and examined using SDS-PAGE, immunoblotting, FT-IR, and AFM. In situ analysis, immunofluorescence (CLSM) and immunogold-labelling (TEM), were performed. We demonstrated that AGPs were indeed present in apple fruits at the different stages of the ripening process. The changes in the amount (1.52–2.08 mg g^−1^), diameter (152.73–75.05 nm), molecular mass (50–250 kDa), and distribution in the cell of AGPs demonstrate their variable presence and changeable structure during the ripening process. We propose specific wavenumbers, i.e. 1265 cm^−1^, 1117 cm^−1^, and 960 cm^−1^, which could be assigned to AGPs. The immunofluorescence and immunogold-labelling results indicate that the JIM13 antibody is the most characteristic for AGPs in apple fruits. This study quantitatively demonstrated for the first time that AGP accumulation occurs in ripe fruits, which is supported by the highest AGPs content, the highest molecular mass, and the appearance of a specific distribution pattern at the cellular level.

## Introduction

Fruit ripening is a complex process in the final phase of fruit development. Dynamic changes in the composition of apple tissue during on-tree maturation, over-ripening and postharvest senescence are the subject of worldwide studies. The timing of the ripening stages is linked with biochemical transformations in the cell wall. These include progressive depolymerisation of the polysaccharide matrix at the beginning of the ripening, changes in the carbohydrate composition such as an increase in the pectin demethylesterification rate, and loss of galactan tightly associated with cellulose microfibrils correlated with rapid softening during postharvest ripening^1^. The content of the cell wall components and their spatial distribution in the fruit cell wall are precisely associated with the stage of development and the ripening process. While changes in cellulose and hemicellulose are less pronounced, the rearrangement of pectins in the cell wall is closely connected with the ripening process^2^. However, not only changes in the polysaccharide structure but also the disassembly of their connections with other components are responsible for the alterations in the cell wall^3^. Other cell wall components associated with fruit ripening are proteins, which play various functional roles in the course the whole fruit maturation. Proteins are related to metabolic changes in the content of soluble solids, respiration, and reduction in firmness during fruit softening by involvement in the destabilisation of the actin cytoskeleton^4^. Interestingly, the presence of arabinogalactan proteins (AGPs), i.e. a type of structural proteins of the cell wall, is closely correlated with subsequent ripening stages^5^, and their spatio-temporal distribution in a particular part of a fruit provides evidence for their role in this process^6^.

AGPs are described as extensively glycosylated hydroxyproline-rich cell wall glycoproteins consisting mainly of arabinose and galactose-rich polysaccharide units attached to diverse protein core sequences^7^. Their molecules are attached to microdomains in the plasma membrane via GPI anchors, which ensures a continuum in the extracellular matrix at the border between the cell wall and the plasma membrane^8^. The structural functions are also a result of the covalent linkage between cell wall polymers in which matrix polysaccharides, i.e. pectin and xylan, are linked to AGP. The presence of the APAP1 complex indicates the role of AGPs in the cross-link between cell wall polysaccharides and glycoproteins. It is assumed that the appearance of APAP1 has consequences in the cell wall arrangement and functions, including structural support, signalling, and cell–cell adhesion. It is also believed that the unique set of structural features of AGPs, mainly the highly branched carbohydrate domains and especially their heterogeneity, are linked to their functions in multiple aspects of plant growth and development^9^.

The knowledge of AGPs found in fruits is based on reports on AGP expression in *Solanum lycopersicum*^10^, *Vitis vinifera*^11^, and *Malus* × *domestica*^12^. Studies on the AGP expression patterns indicated that they are regulated in the tomato ripening developmental programs. Moreover, a higher level of AGPs showed their participation in the rapid response to mechanical wounding and thus their involvement in defence against abiotic stresses^10^. In the case of grapes, AGPs were present in a significantly higher amount in ripe fruits. The molecular probe, i.e. the LM2 monoclonal antibody recognising specific carbohydrate moieties of AGPs, was identified as a biomarker of grape ripening^11^. Immunocytochemical approaches used to examine changes in the spatio-temporal-dependent localisation of AGPs in the apple fruit cell wall at the cellular level showed their presence in different zones, depending on alterations in the cell wall-plasma membrane during the ripening process. In addition, the distribution of AGPs in *Malus* × *domestica* fruits depends on the condition of the cell wall-plasma membrane, which changes along with the senescence process during postharvest storage or upon fungal infection^12^.

Determination of factors implicated in ripening opens new research avenues for controlling fruit quality. Strikingly, despite the huge interest, no detailed research has been done so far on AGPs found in fruits, and their content, role, and structure in fruits during ripening are unknown. In the present work, AGPs in apple fruits during different stages of ripening are characterised for the first time. For this purpose, in situ and ex situ analyses were performed. Scientific tools, i.e. such immunocytochemistry methods as immunofluorescence labelling (CLSM) and the immunogold-labelling method at the subcellular level (TEM) with antibody-based probes, gave a possibility to confirm the AGP presence *in planta.* Moreover, the research consisted in identification of AGPs isolated from apple fruit tissue. We used the most characteristic criterion in recognition of AGPs, which is also essential in the extraction procedure, i.e. a specific AGP-disrupting agent—the β-glucosyl Yariv reagent (β-GlcY). The quantitative and qualitative analysis of AGPs found in the fruits was performed using molecular approaches, such as SDS-PAGE and immunoblotting. The use of numerous monoclonal antibodies recognising various antigenic determinants facilitated comparative analysis of the dynamic changes in AGP molecular mass during ripening. In addition, for comprehensive structural analyses of the AGPs, FT-IR spectroscopy and atomic force microscopy studies were performed. The results obtained not only demonstrate the feasibility of these approaches in AGP structural analyses, but also provide new evidence for the function of carbohydrate residues in AGPs with respect to the fruit ripening process.

## Results

### Protein quantification in apple fruits at different stages of ripening

**Figure 1:**
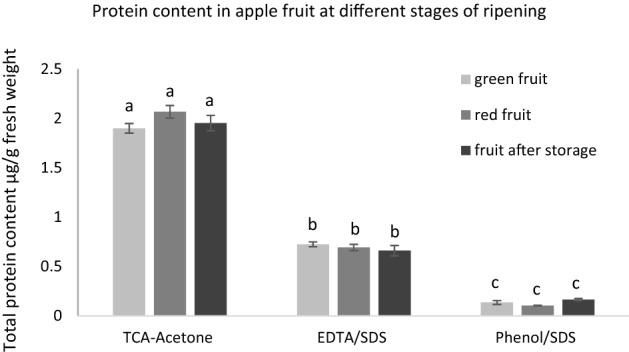
Qualitative analysis of total protein content in green, red and stored apple fruit extracted by using three different protocols: TCA/acetone precipitation with phenol extraction, EDTA/SDS extraction and phenol/SDS extraction. Bars show the standard error. Different letters indicate significant differences among the development stages (according to ANOVA with *p* < 0.05).

As shown in Fig. 1, protein yield from apple flesh does not depend on the ripening stage. However, the protein yield depends on extraction protocol. TCA-Acetone precipitation with phenol extraction was the most effective, resulted in the protein content approximately 2.1 (± 0.09) µg g^−1^ of fresh weight while the phenol/SDS and EDTA/SDS extraction methods yielded a significantly lower amount of protein, 0.13 (± 0.02) µg g^−1^ and 0.69 (± 0.04) µg g^−1^ respectively).

### Qualitative analysis of AGPs extracted from fruits at different stages of ripening

**Figure 2:**
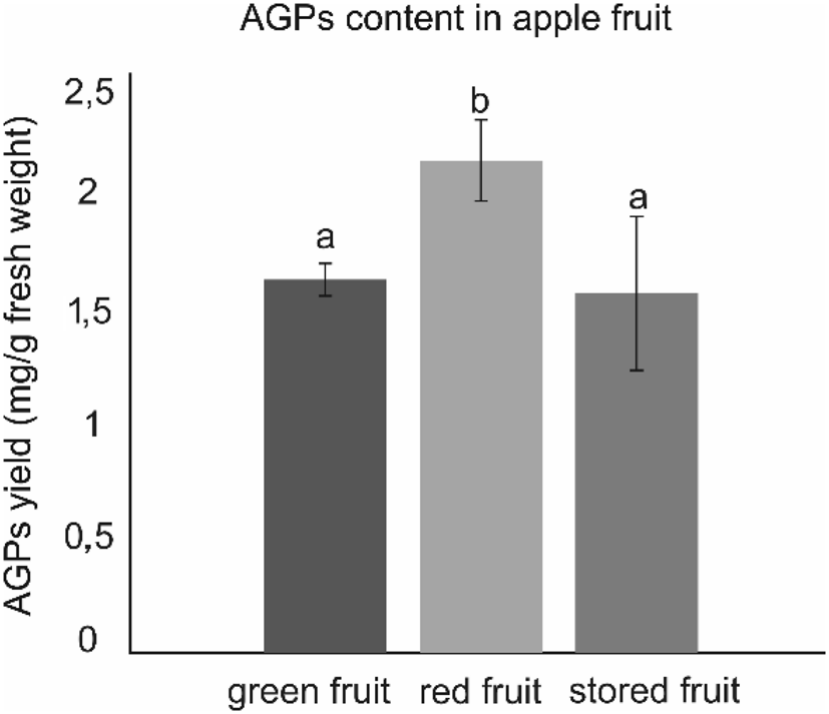
AGPs content in green, red and stored apple fruit: mg g^−1^ of fresh weight of parenchymal tissue. Bars show the standard error. Different letters indicate significant differences among the development stages (according to ANOVA with *p* < 0.05).

The arabinogalactan protein isolation using the Yariv reagent method carried out as in Lamport studies^13^ confirmed the changeable amount of AGPs during the ripening process (Fig. 2). Significantly higher amount of precipitated AGPs was obtained in the extract from red fruit (2.08 ± 0.17 mg g^−1^ of fresh weight). In the green and stored apples concentration of AGP was similar, i.e. 1.58 (± 0.06) mg g^−1^ and 1.52 (± 0.32) mg g^−1^ of fresh weight, respectively.

### Molecular masses of AGPs—SDS-PAGE and immunoblotting

**Figure 3:**
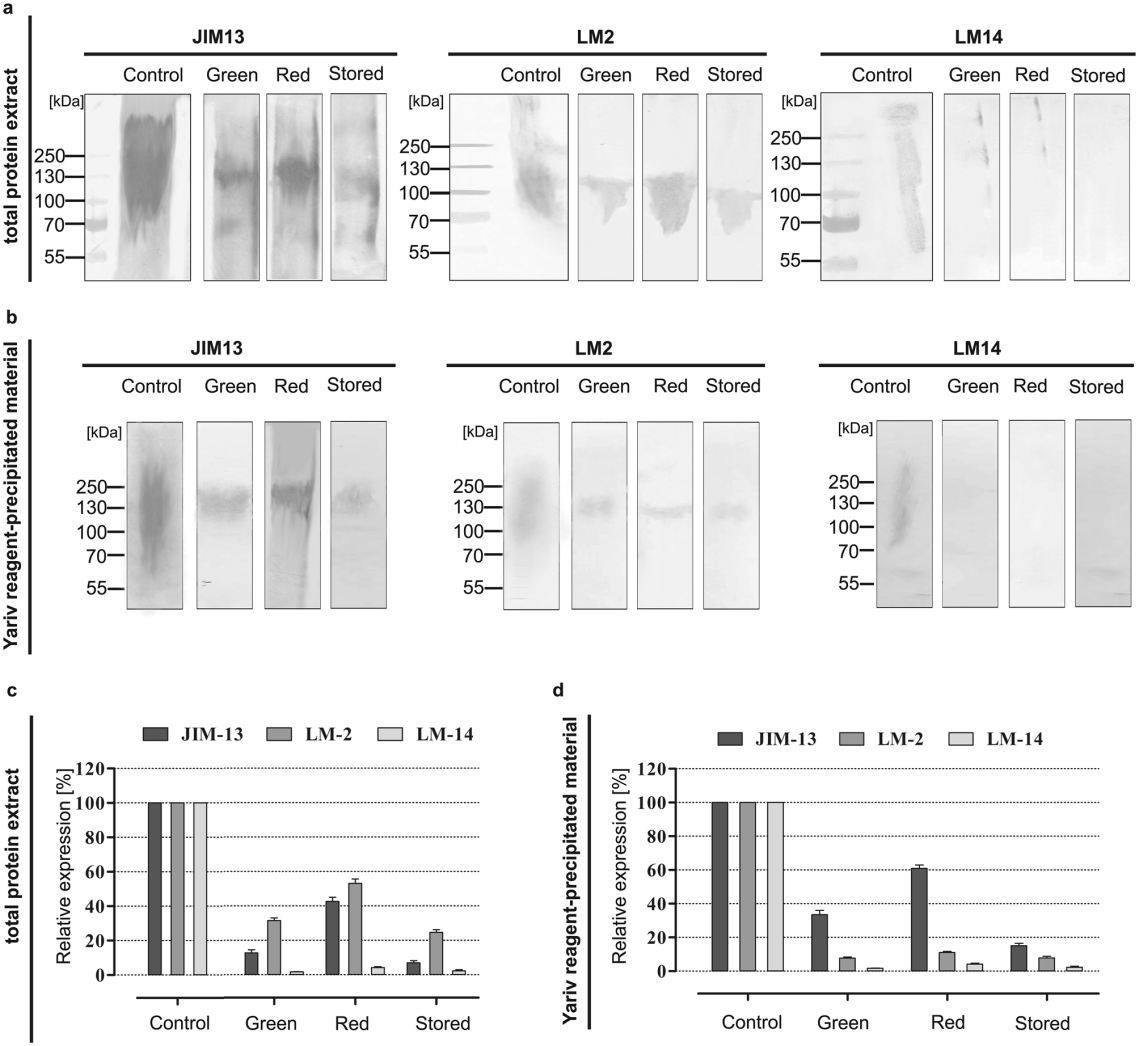
Immunoblotting of AGPs in green, red and stored apple fruit. Total protein extract (**a**) and Yariv reagent-precipitated material (**b**) were separated in SDS-PAGE for immunoblot analysis with JIM13, LM2, and LM14. Immunoblotting relative expression [%] of AGPs in total protein extract (**c**) and Yariv reagent-precipitated material (**d**). Expression level was estimated by densitometric measurements of obtained stripes by ImageJ 1.51 software. Molecular masses are indicated on the left. The samples derive from the same experiment and blots were processed in parallel.

Two types of material: the total protein extract (Fig. 3a) and Yariv reagent-precipitated material (Fig. 3b) were subjected to SDS-PAGE and protein immunoblotting. Gum Arabic was used for comparison and as a positive control.

In both types of material the molecular masses of AGPs from apple fruits in the three examined stages of ripening were dispersed. Examination of the total protein extract from the fruits showed changes in the molecular masses of AGPs detected by JIM13 depending on ripening stage: from 70 to 200 kDa in the green fruit, from 70 to 250 kDa in the red fruit, and from 50 to 130 kDa in the stored fruit. AGPs detected by LM2 resulted in the molecular mass in the range of 60–120 kDa in the green and red fruits as well as 60–100 kDa in the stored fruits. AGPs with a molecular mass of 100 kDa, 130 kDa and 250 kDa were recognised by LM14 in the green fruit samples and 130 kDa, 250 kDa in the red fruit. The LM14 labelling of an equivalent gel did not detect AGPs in the extract from the stored fruits. Immunoblotting relative expression analyse also confirmed changeable detection of AGPs recognized by different antibodies (Fig. 3c).

Yariv reagent-precipitated material was purified by dialysis; thus, it contained relatively more homogeneous AGP molecules, which were visible as a single band on immunoblots probed with anti-AGP antibodies (Fig. 3b). The analysis with JIM13 showed alterations in the content of JIM13 epitopes during the ripening stages, with a significant decrease in the AGP molecular mass in the stored fruits (Fig. 3d). AGPs with a molecular mass of 120–250 kDa were recognised in the red fruits, while AGPs with molecular mass 100 and 120 kDa were detected in the stored material. AGPs with a molecular mass of 120 kDa were recognised by LM2 only in the red fruit samples. Interestingly, the LM14 probe did not detect AGPs from all examined stages.

### In situ analysis of AGP presence in fruits during the ripening process

**Figure 4:**
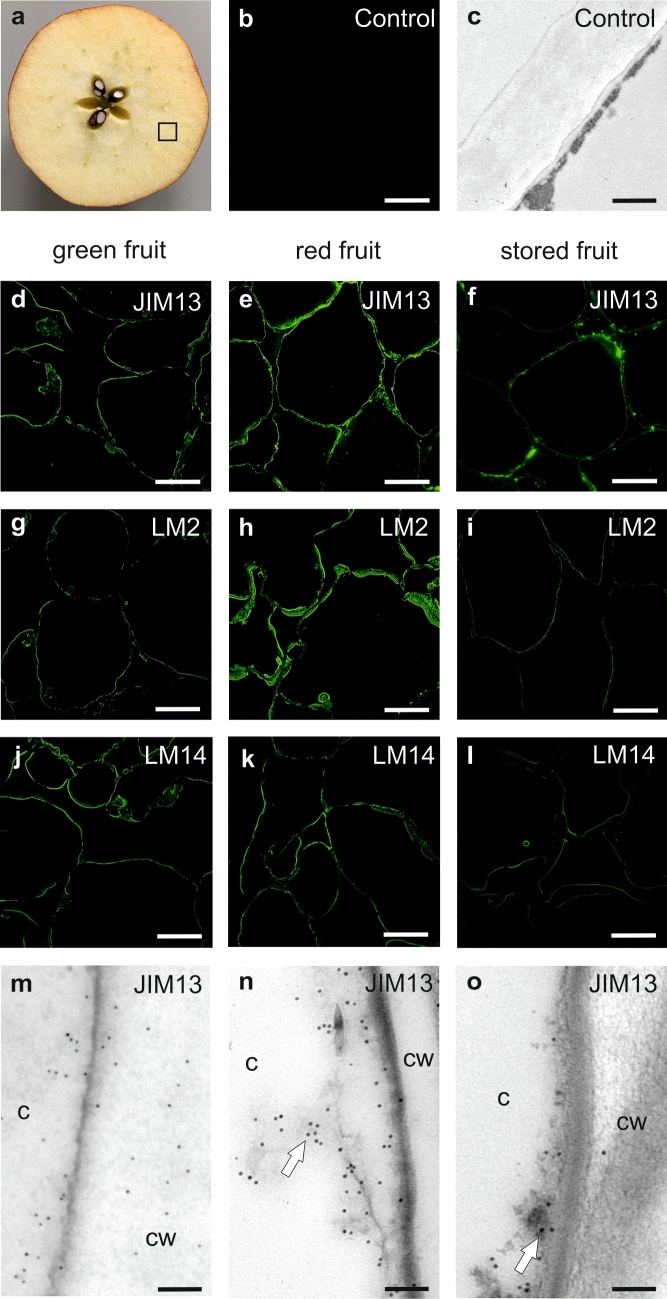
Cellular and subcellular immunolocalization of AGPs in parenchymal cells of green, red and stored apple fruit (**a**), imaging using CLSM and TEM. Control reactions (**b** and **c**). Immunofluorescence of JIM13 epitope in green fruit (**d**), red fruit (**e**) and stored fruit (**f**). Immunofluorescence of LM2 epitope in green fruit (**g**), red fruit (**h**) and stored fruit (**i**). Immunofluorescence of LM14 epitope in green fruit (**j**), red fruit (**k**) and stored fruit (**l**). Immunogold-labelling of JIM13 epitope in green fruit (**m**), red fruit (**n**) and stored fruit (**o**). Bars: 50 µm for CLSM, and 500 nm for TEM. *c* cytoplasm, *cw* cell wall.

Immunocytochemical analyses in situ were performed to confirm the presence of AGPs in the apple fruits in the evaluated ripening stages. Cube-shaped parenchymal tissue was examined using immunofluorescence and immunogold-labelling methods with specific monoclonal antibodies against AGP epitopes (Fig. 4a). As a control, labelling without primary antibodies was performed in both techniques and imaged with CLSM (Fig. 4b) and TEM (Fig. 4c). No labelling was observed in the control samples, which confirmed the specificity of both methods.

Labelling with the JIM13 antibody showed the presence of the β-GlcA-(1 → 3)-α-GalA-(1 → 2)-α-Rha epitope in the fruit cell wall (Fig. 4d–f). The most characteristic pattern of AGP distribution was observed in the red fruits, where JIM13 epitopes were significantly labelled as a line at the border of the cell wall (Fig. 4e). The localisation of AGPs in the green (Fig. 4d) and stored (Fig. 4f) fruits was different than in the red fruit samples, and the fluorescence intensity was lower. The LM2 epitope was also present in the fruit tissue in all examined ripening stages (Fig. 4g–i). However, fluorescence was most visible in red fruit samples (Fig. 4h), in comparison to the green (Fig. 4g), and stored fruits (Fig. 4i). The use of the LM14 antibody, which recognises arabinose- and galactose-enriched units of the carbohydrate motif of AGPs, confirmed the presence of the AGPs epitope in the analysed fruit cells, but in a substantially lower amount than that shown by the other antibodies (Fig. 4j–l).

The immunogold-labelling revealed the specific spatial distribution of AGPs in the fruit cells. JIM13 epitopes, visible as dark points, were observed throughout the cell wall, and labelling occurred preferentially close to the wall region near the plasma membrane in the red fruits (arrows in Fig. 4n,o). This pattern was disrupted in the green fruits, where AGPs were dispersed in the whole cell wall (Fig. 4m). Similarly, in the stored fruits, AGP epitopes in damaged cell walls were detected less frequently, i.e. only in single points along the cell wall-plasma membrane continuum (Fig. 4o).

### Structure of AGPs—FT-IR spectroscopy

**Figure 5:**
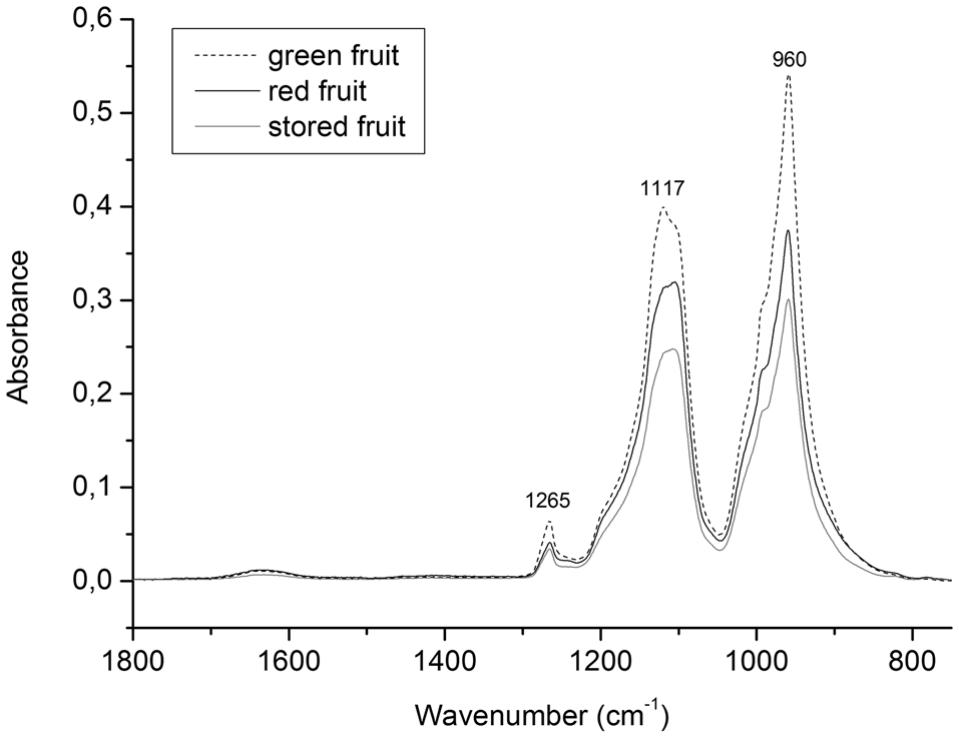
FT-IR spectra of AGPs extracted from green, red and stored apple fruit in the 1800–800 cm^−1^ region.

Figure 5 presents the FT-IR spectra in the range of 1800–750 cm^−1^ of the examined AGP material extracted from fruits in the three different stages of ripening. The results indicate that the 1300–950 cm^−1^ region is characteristic for AGPs. Three bands at 1265, 1117, and 960 cm^−1^ provided additional evidence to support the presence of AGP units in the fruit material. The peak at 1265 cm^−1^ is attributed to C–O stretching vibrations in acidic pectin^14,15^, and is characteristic for the amide band in proteins^16^. The peak at 1117 cm^−1^ belongs to uronic acids in pectic polysaccharides^17^, and that at 960 cm^−1^ indicates the presence of arabinogalactan, as described earlier by Robert et al.^18^.

Additionally, there were differences in the intensity of peaks between the examined stages, with a decrease in the intensity of the bands along the ongoing process of ripening.

### Nanostructure of AGPs—AFM imaging

**Figure 6:**
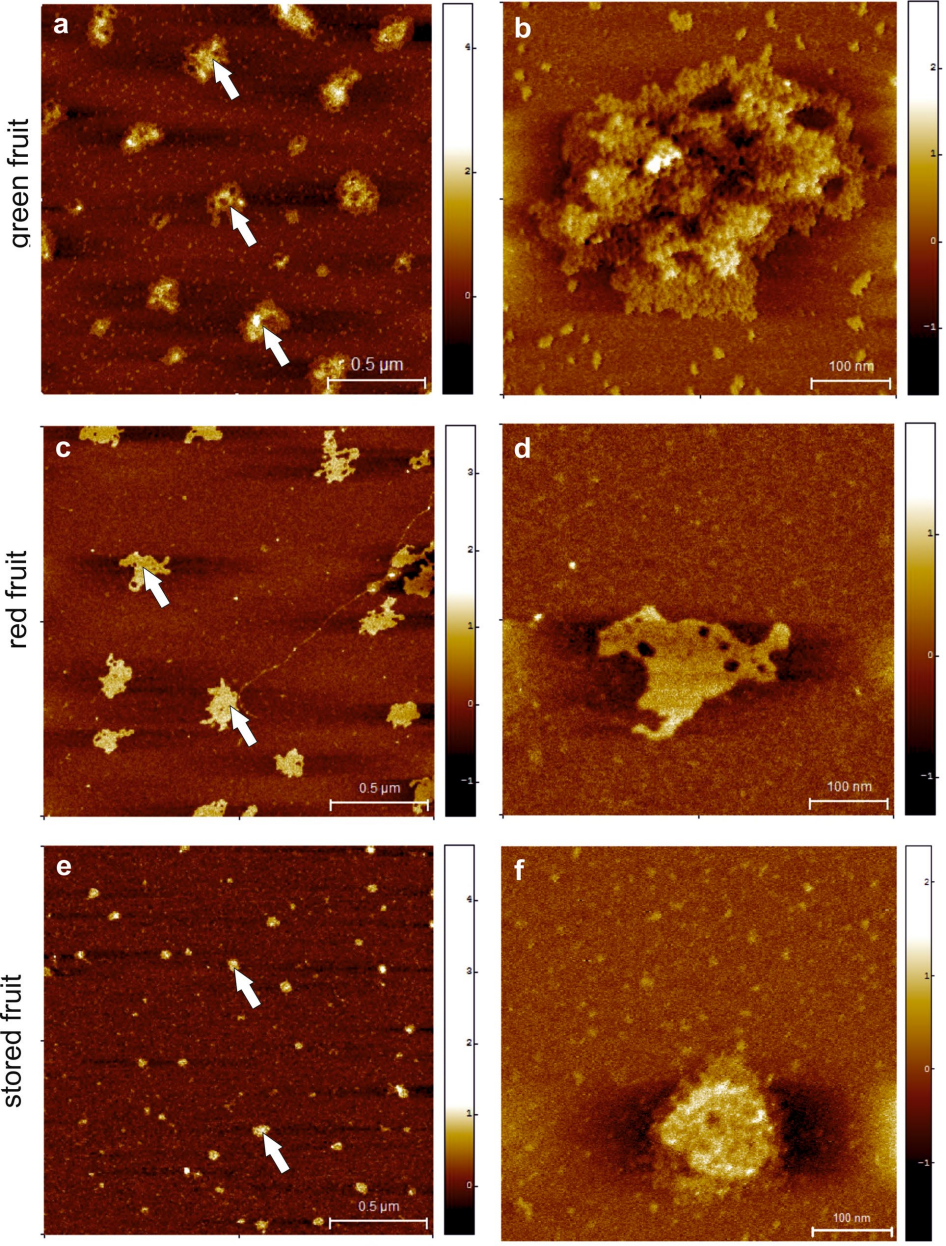
AFM imaging of AGPs extracted from green (**a**, **b**), red (**c**, **d**) and stored apple fruit (**e**, **f**). Particular aggregates marked by arrows. Bars: 0.5 µm (**a**, **c**, **e**) and 100 nm (**b**, **d**, **f**).

Representative AFM images of AGPs isolated from fruits in the different stages of ripening and deposited on mica are shown in Fig. 6. For each analysed sample two classes of objects were observed, i.e. larger packed aggregates and numerous significantly smaller individual molecules (aggregates marked by arrows on Fig. 6a,c,e). The aggregates in the samples of AGPs extracted from the green (Fig. 6b) and red (Fig. 6d) fruits had a more irregular extended shape, while the aggregates in the sample of AGPs from stored fruits (Fig. 6f) were more compact.

Table 1 presents average values with standard deviations of the geometric parameters of AGP molecules from AFM images. On average, the AGP molecules extracted from the green apple fruits were significantly larger than those in the red and stored samples. The mean diameter of the green sample particles was two-fold larger (152 nm) than two other samples (about 80 nm) and their area was respectively 3–4 times larger than in the case of the red and stored samples. The AGPs molecules from the green fruits were also considerably higher (0.9 nm). The analysis of the shape parameters (aspect ratio, compactness and elongation) has shown that only AGPs from the stored fruit differs from the other samples. Objects for the stored samples became less elongated (elongation for circle is 0) and more regular in shape (compactness for circle is 1).

**Table 1:** Average values with standard deviations of geometric parameters of AGPs molecules deposited on mica.

	Diameter (nm)	Area (nm^2^)	Maximum height (nm)	Mean height (nm)	Aspect ratio	Compactness	Elongation
Green apple fruit	152.73 ± 65.91^a^	21,729.36 ± 16,702.44^a^	1.92 ± 0.64^a^	0.90 ± 0.25^a^	1.38 ± 0.30^a^	0.77 ± 0.09^a^	0.25 ± 0.13^a^
Red apple fruit	88.80 ± 39.63^b^	7424.94 ± 6674.01^b^	1.34 ± 1.37^b^	0.68 ± 0.32^b^	1.37 ± 0.36^a^	0.78 ± 0.11^a^	0.24 ± 0.15^a^
Stored apple fruit	75.05 ± 24.52^c^	4895.17 ± 3225.36^c^	1.36 ± 0.78^b^	0.75 ± 0.28^c^	1.25 ± 0.24^b^	0.82 ± 0.08^b^	0.18 ± 0.11^b^

The same superscript letters mean absence of significant differences (according to ANOVA with p = 0.05).

## Discussion

The ripening process is a repeatedly undertaken subject of research. Most investigations are focused on the major components of the cell wall, i.e. polysaccharides, which is understandable given their content and functions. However, there is a gap in the knowledge of cell wall components present in smaller amounts, which cannot exclude their great importance for the functioning of the whole extracellular matrix as a network of structural interdependencies. Studies on validation of universal protocols for protein extraction demonstrated that the average protein content in apple flesh was approximately 291 µg g^−1^ and 94.5 µg g^−1^ of fruit fresh weight^19,20^. For the best method we obtained less than 2.5 µg g^−1^ of fruit fresh weight that demonstrates that apple fruits are a low protein source, regardless of stages of ripening. However in this work we focused on the precise content of one type of structural proteins—AGPs. For this purpose, the extraction procedure with additional steps based on the use of the specific Yariv reagent was performed. It has been previously established that the target structure for the β-Gal-Yariv reagent is the β-1,3-galactan chains longer than five residues, which are conserved carbohydrate structures of the AGP molecule^21^. Moreover, for efficient cross linkage by the Yariv reagent, the polysaccharide moiety must be templated by attachment to a polypeptide^13^. Quantification of AGPs in plant tissue was carried out by Lamport et al.^13,22^ with strong emphasis on the differentiation of the AGP amount in various plant cell compartments, bounded to the outer surface of the plasma membrane by a GPI anchor, trapped in the cell walls and soluble in the periplasm^13^. Studies on tobacco BY-2 cells indicated the highest AGP content in the periplasm after cell rupture and the lower content of AGPs in the plasma membrane and cell wall. These results showed the total AGP concentration was around 600 µg g^−1^ of tobacco cells^13^. In our work on apples, the AGP concentration in the parenchyma tissue was 2.08 (± 0.17) mg g^−1^ of red fruit fresh weight. In comparison to the presence of AGPs in other plant tissues, the concentration in the apple tissue was three-fold higher, which confirms that the apples are a good source of the examined proteoglycans although the total protein content in low. On the other hand, the extraction method using the Yariv reagent yields material with precisely specified characteristic features. This conclusion lead to the next aim of the current work that was to determine the properties of AGPs extracted from fruits and describe their possible changes as a result of the progressing ripening process.

In previous studies, substantial changes in the AGP content in different stages of the ripening process were observed^11^. Antibodies directly recognising AGPs provide information about the presence of specific AGP epitopes in extracts, which facilitates determination of the AGPs profile during the ripening process. The use of LM2 antibodies elicited the strongest response in grape berries and showed an increase in AGP epitope abundance in the ripe stage^11^. Similarly, in case of tomato pericarp tissue, the expression of a class of AGPs, i.e. SlAGP2, was significantly higher at the turning stage of ripening. Moreover, the AGPs from tomato pericarp comprised the JIM13 epitope ranging from molecular mass of 45–300 kDa. The immunoblotting revealed no alterations in the high molecular weight AGPs during ripening, but only a slight decrease in the AGP expression in red ripe tomato fruits was detected^10^. Our present quantitative studies of the content of AGPs in apple fruits from the green unripe stage (“green”), through mature fruit (“red”), to fully ripe post-storage (“stored”) fruit showed changes in their amount as well as their structural properties. AGP-like smeared bands in a high molecular weight range were observed on SDS-PAGE gel stained by antibodies that commonly used for specific labelling of glycan epitopes of typical AGPs. The broad bands of AGPs were present in the total protein extract and in the Yariv reagent-precipitated material but appeared considerably stronger in the total protein extract with apparent AGP molecular mass in a wide region from 70 to 250 kDa. In the case of the Yariv reagent-precipitated material, the fraction of AGP molecules was more homogenous and appeared in a more precise region. This shows that the method for AGP extraction requires the use of Yariv reagent. Furthermore, the immunoblotting of both types of material indicated that JIM13 was the most effective antibody for AGPs in the fruits. The other antibodies used, i.e. LM2 and LM14, bound AGPs as well, but less effectively than JIM13. It should be underlined that the strongest signal shown by the immunoblotting analysis was detected in AGPs from the red fruits, in comparison to other examined stages. In agreement with this result, the immunofluorescence and immunogold-labelling indicate that JIM13 epitopes occurred with a specific pattern for AGPs also at this stage. This may correspond with the hypothesis that the JIM13 antibody is the most characteristic for AGPs in apple fruits in the mature ripe stage of development. It can therefore be used as a marker of this ripening stage. All analyses performed in the present study showed that AGP accumulation occurs in this stage of ripening, which is supported by the highest AGPs content, the highest molecular mass, and the appearance of specific distribution pattern at the cellular and subcellular level.

Infrared spectroscopy combined with Fourier transform (FT-IR) is commonly applied for monitoring cell wall components and changes therein during processing^15^. The first information about fingerprinting of AGPs was provided for AGPs extracted from green tea leaves^16^. The analysis of the whole region of 4000–400 cm^−1^ divided into four subregions revealed that the 1200–800 cm^−1^ region was the most representative for AGPs. It is also commonly used in investigations of polysaccharides present in higher plants. Also, the proteins are characterised by amide bands between 1720 and 1600 cm^−1^, 1600 and 1500 cm^−1^, 1450 and 1200 cm^−1^^16^. In our work the FT-IR results highlighted the characteristic wavenumbers of AGPs extracted from apple fruits. The infrared absorption spectrum of AGPs had a unique fingerprint region from 1300 to 950 cm^−1^. Due to carried out the analysis of the specific positioning and intensity of bands on FT-IR spectra of AGPs, the present work proposes identification of specific wavenumbers that can be assigned to AGPs. The spectra of AGPs extracted from the apple fruits in the different stages of ripening have similar bands denoting AGPs at 1265 cm^−1^, 1117 cm^−1^, and 960 cm^−1^. Two of the examined bands are sharper and more intensive. They correspond to arabinogalactan (960 cm^−1^) and uronic acids (1117 cm^−1^), which are the main components of the carbohydrate chains of AGPs. According to Coimbra et al.^17^ broad band at 960 cm^−1^ can be the effect of overlapping bands characteristic for arabinose and galactose units, which the main absorbance regions are 1060–1040 and 975 cm^−1^ (arabinose) and 945 cm^−1^ (galactose). Moreover, the band at 1265 cm^−1^ is significantly less intensive, which may be related to presence protein. Interestingly, the intensity of the same bands between various AGPs extracted from apple fruits in the different stages of ripening varies. All peaks have the highest value in AGPs from the green apple fruits and the lowest value in AGPs extracted from the stored fruits. Taken together, the results demonstrate that the AGP structure is changeable during the ripening process. It has also been shown that FT-IR analysis is an effective tool for differentiation of the AGPs obtained from the fruits and for monitoring changes in their structure during different processes.

The light scattering analyses of AGPs from gum Arabic revealed that their molecular diameters were approximately 80 nm. The AGP component was characterised by an elongated structure, with thin linear sections and linear arrays of globules, which was consistent with the ‘wattle blossom’ model of the AGP molecule. The elution profile obtained by SEC-MALS showed a mixture of small globular features and more elongated structures, which were visible as aggregates of globules^23^. A high-resolution study of enhanced green fluorescent protein (EGFP)-labelled LeAGP1 extracted from *Arabidopsis thaliana* using atomic force microscopy showed a strong tendency of AGPs to form clusters, arcs, and branched rings called ‘nanopores’. The authors suggested that various structural units of AGPs are dependent on the type of the cell and on the position in the cell. Moreover, the presence of a distinctive structural form is related to the developmental stage and stressful circumstances. This tendency is correlated with the structure of AGP macromolecules, situating the carbohydrate moieties outside the molecule and the protein part inside^24^. On the other hand, nanoparticles of AGPs isolated from *Hedera helix* were approximately 70 nm in diameter. They were predicted to be spheroidal according to ‘wattle-blossom’ model rather than clusters of multiple molecules^25^. As described in a previous study^7^, AGPs exhibit a capacity to agglomerate, which corresponds with their physiological interactions, i.e. as a predominant component of sticky exudates in *Acacia* and *Hedera helix.* In *Hedera helix*, the progress of the AGP molecules agglomeration process was associated with the formation of a compact biofilm with significant adhesive properties^25^. Similarly, AGPs as a major fraction of gum Arabic were described as elongated aggregated structures as well as a more or less anisotropic spheroidal shape single particles. Observed AGPs conformations are effect of heterogeneity in the degree of AGPs branching, which is also reflected in the asymmetric molecular weight distribution and large size polydispersity of side chains^26^. As shown in our work, the size of AGPs extracted from the apple fruits varied in the different stages of ripening, with a pronounced decrease in the AGP size in the stored fruits. The mean size of aggregates was from 75 to 150 nm that is consistent with previous study. Additionally, the shape was modified and the tendency towards aggregation was lower during ripening process. We hypothesise that the formation of aggregates is correlated with the presence of carbohydrate chains in the AGP molecule. As it has been already demonstrated, with the progress of the ripening process, the amount of arabinogalactan and uronic acids, i.e. the predominant constituents of AGP carbohydrate chains may decrease. This is in line with lower intensities of FT-IR bands of AGPs from the stored fruits, a weaker or absent fluorescence signal in immunofluorescence labelling, a lower amount of gold particles in immunogold-labelling, and substantially lower molecular mass determined by immunoblotting. The antibodies used recognise epitopes composed of arabinose, galactose residues, and glucuronic acid. The content of the epitopes was disrupted in the AGPs isolated in this stage, which was demonstrated by all the immunocytochemical techniques employed. We postulate that the proper structure of AGP carbohydrate chains is indispensable for formation of the typical structure of AGPs. This may indicate that loss of side branches or stronger entangling of polymers induces occurrence of additional intermolecular interactions between carbohydrate moieties and polypeptide backbone during the ripening process.

Overall, the results obtained are in agreement with reports on the impact of AGPs on cell wall arrangement. Prolyl 4 hydroxylases (P4Hs) catalyse proline hydroxylation, i.e. a major post-translational modification of cell wall hydroxyproline-rich glycoproteins (HRGPs), including AGPs, and thus influence their proper structure and function. In fruits with disturbed function of P4Hs, the total hydroxyproline content is lower and disturbances in the AGP structure lead to structural and functional changes during cell division and expansion^27^. All posttranslational modifications, which include conversion of proline residues into hydroxyproline by P4H, have an impact on the covalent network in the cell wall. Therefore, the correct glycosylation of AGPs is essential for proper cell wall assembly^28^. In our studies, we observed changeable features of the AGP structure, mainly the carbohydrate chains, in the native process at the cellular and molecular level. Based on this, we may suppose that in the case of fruit the condition of the AGP carbohydrate moiety is most important for proper formation of the AGP molecule, which is the basis for accurate functioning of AGPs in the plant cell.

To sum up, the analysis described in this paper demonstrated the presence of AGPs isolated with use of Yariv reagent in the parenchyma tissue in the apple fruits in different stages of the ripening process. The in situ and ex situ studies revealed compatible results. The changes in the amount (1.52–2.08 mg g^−1^), diameter (152.73–75.05 nm), molecular mass (50–250 kDa), and distribution in the cell (in the cell wall-plasma membrane or dispersed on the whole cell wall) of AGPs confirmed the variable presence and changeable structure of AGPs in fruits during the ripening process.

## Materials and methods

### Plant material

Apple fruits cv. ‘Jonaprince’ were used for experiments. The fruits were collected from a local producer (Lublin, Poland), approximately a month before the optimal harvest date at the onset of fruit ripening (called as “green” apple fruits) and immediately after the historical harvest date for these cultivars at the optimum maturity (called as “red” apple fruits). The red apple fruits were stored in a cold room at 4 °C in a normal atmosphere for 1 month (called as “stored” apple fruits).

### Protein extraction

**Figure 7:**
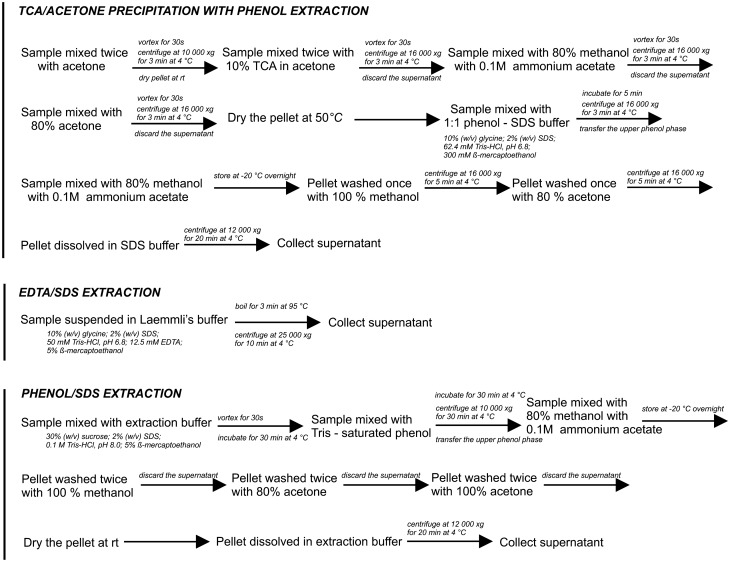
Preparation of protein samples using three methods: TCA/acetone precipitation with phenol extraction, EDTA/SDS extraction and phenol/SDS extraction.

Samples were taken from the parenchyma tissue, next were frozen, homogenised, and processed for protein extraction. Total proteins were extracted for all ripening stages in triplicate. Three different protein extraction methods were used: TCA/acetone precipitation^19^, EDTA/SDS extraction^10^, and phenol/SDS extraction^4^. The schematic flow of the main steps of these methods is shown in Fig. 7. The protein content in each sample was determined with the colorimetric Bradford assay using bovine serum albumin (BSA) as a standard^29^.

#### TCA/acetone precipitation with phenol extraction

Samples with identical weight were resuspended in acetone twice and centrifuged at 10,000 × *g* at 4 °C for 3 min. The resultant pellet was transferred into a mortar, allowed to dry at room temperature, and ground to a fine powder. The samples were treated with 10% TCA in acetone and centrifuged at 16,000 × *g* at 4 °C for 3 min. The step was repeated until the supernatant was colourless. The obtained pellet was washed with 80% methanol containing 0.1 M ammonium acetate, vortexed and centrifuged, and the supernatant was discarded. The washing procedure was repeated with 80% acetone, and the pellet was dried at 50 °C to remove residual acetone. Then, the tubes with pellets were filled with 1:1 phenol/sodium dodecyl sulphate buffer (62.5 mM Tris–HCl, pH 6.8.; 2% (w/v) SDS; 10% (v/v) glycine; 300 mM β-mercaptoethanol) to extract proteins from the dry pellet, incubated for 5 min, and centrifuged at 16,000 × *g* for 3 min. The upper phenol phase was pipetted and precipitated with five volumes of 80% methanol containing 0.1 M ammonium acetate and stored at − 20 °C overnight. Subsequently, the white pellet was washed once with 100% methanol and then once with 80% acetone. During each wash step, the tube was centrifuged at 16,000 × *g* at 4 °C for 5 min. The final pellet was dissolved in SDS buffer.

#### EDTA/SDS extraction

Fruit samples were homogenised with modified Laemmli’s buffer containing 50 mM Tris–HCl, pH 6.8, 2% (w/v) SDS, 710 mM β-mercaptoethanol, and 12.5 mM EDTA. The homogenates were boiled for 3 min and clarified by centrifugation at 25,000 × *g* at 4 °C for 10 min.

#### Phenol/SDS extraction

Frozen ground fruit samples were suspended in extraction buffer. After incubation at 4 °C for 30 min, an equal volume of Tris-saturated phenol was added, and the mixture was centrifuged at 10,000 × *g* at 4 °C for 30 min. The upper phenol phase was collected and precipitated with 0.1 M ammonium acetate in methanol overnight at − 20 °C. The pellet was rinsed twice with methanol, twice with 80% acetone, and once with 100% acetone. After that, the pellet was air dried at room temperature and dissolved in the extraction buffer. The supernatant was collected after centrifugation at 12,000 × *g* at 4 °C for 20 min.

### Isolation of AGPs from apple fruits in different stages of ripening

**Figure 8:**
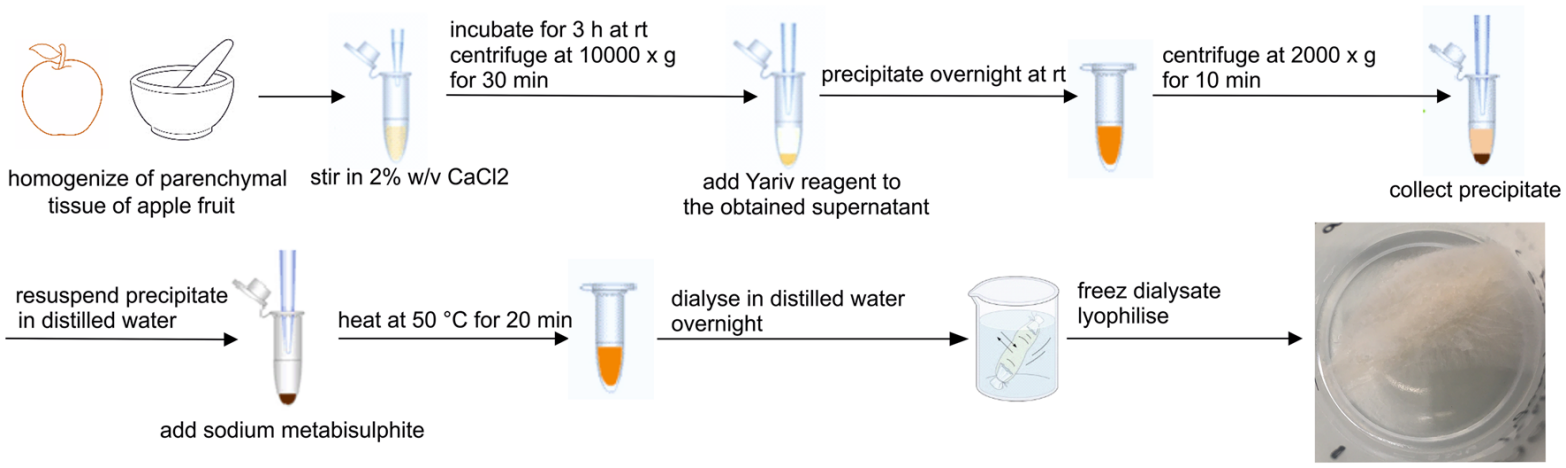
Schematic protocol of AGPs isolation from fruit tissue. Scheme was edited using the CorelDrawX6 graphics program.

AGPs were isolated according to an earlier protocol^22^ based on the selective precipitation of AGPs with the β-D-Glucosyl Yariv reagent (Biosupplies, Australia), with some modifications. The schematic protocol of these method is shown in Fig. 8. Briefly, frozen fruit tissue was homogenised and stirred in 2% (w/v) CaCl_2_ at room temperature for 3 h. The homogenate was centrifuged at 10,000 × *g* for 30 min and the supernatant was retained. AGPs were precipitated with the Yariv reagent by mixing the supernatant with an equal volume of β-GlcY (1 mg mL^−1^) in 2% (w/v) CaCl_2_ and left overnight at room temperature. The precipitate (insoluble Yariv-AGP complex) was collected by centrifugation (15 min at 2000 × *g*) and dissolved in MiliQ water. For reduction of the diazo linkage, sodium metabisulphite was added gradually and heated at 50 °C until the red colour disappeared. After cooling, the resulting clear yellow solution was transferred to dialysis tubing with a 12-kDa MW cutoff (32 mm flat width, Sigma, USA) and stirred overnight for 24 h. Finally, the dialysate was freeze-dried and weighed.

### Electrophoresis (SDS-PAGE) and immunoblotting

Gel electrophoresis and immunoblotting analysis were performed for chemical identification and sizing of AGPs extracted from the fruit tissue in the three different stages of ripening. The total protein extract after EDTA/SDS extraction and Yariv reagent-precipitated material (10 µg) were dissolved in SDS buffer, and then 25 µl of sample were loaded per lane. Proteins were separated by 8% SDS-PAGE^30^ and transferred onto an Immmobilon P membrane with 0.45 µm pore size (Millipore IPVH00010). A PageRuler Plus Prestained Protein Ladder was used (cat. no. 26619, Thermo Fisher Scientific, USA) for monitoring the proper transfer of protein onto membrane, the progress of protein migration during SDS-electrophoresis and for estimating the approximate size of separated proteins. Immunoblotting of electrophoresed proteins was performed as reported previously^10^ with slight modifications. Immunoblot analysis with JIM13, LM2, and LM14 monoclonal antibodies against AGP glycan chains was performed for identification of the molecular mass of AGPs. The membranes were blocked with 5% low fat milk for 30 min and incubated overnight at 4 °C with primary antibodies at a concentration of 1:100. After three washes with PBS enriched with 0.05% Triton X-100 (Sigma), the membranes were incubated with secondary anti-rat antibodies conjugated with alkaline phosphatase (AP, Sigma) for 2 h at room temperature. Proteins were detected with AP substrates: 5-bromo-4-chloro-3-indolylphosphate (BCIP) and nitro-blue tetrazolium (NBT) (Sigma) in *N*,*N*-dimethylformamide (DMF, Sigma). Gum Arabic (Biosupplies Australia, concentration 1:1000) was the positive control. The results were analysed qualitatively on the basis of the band thickness, width, and colour depth. The quantitative analysis of protein bands was performed by densitometric measurements of obtained stripes using ImageJ 1.51 software. Three independent experiments were performed.

### Primary antibodies against AGPs

Three most commonly used primary monoclonal antibodies JIM13, LM2, and LM14 were chosen for the immunolabelling procedures, both for microscopic methods and for immunoblotting. The epitope of JIM13 has been determined to have the structure β-GlcA-(1 → 3)-α-GalA-(1 → 2)-α-Rha unit of the carbohydrate motif of AGPs^31,32^. The LM2 epitope is reported as the (1 → 6)-β-Gal unit with terminal β-GlcA in AGP, β-GlcA-(1 → 6)-β-Gal-(1 → 6)-β-Gal-(1 → 6)-β-Gal-(1 → 6)^32,33^. LM14 recognises arabinose- and galactose-enriched carbohydrate chains—arabinogalactan (AG) type II^34^. The antibodies used were obtained from Plant Probes—The Paul Knox Cell Wall Lab at the University of Leeds (UK).

### Immunolabelling using confocal laser scanning microscopy (CLSM)

Immunofluorescence labelling using antibody-based probes was performed for in situ observation of the presence of AGP epitopes in the fruit cell wall. Fixation, resin embedding, and thin and ultrathin sectioning, as well as the immunofluorescence technique procedure were performed as described in our previous paper^35^. Methods and detailed clues proposed by Wilson and Bacic^36^ were used to prepare samples for microscopy analysis. The fruit segments were fixed in 2% (w/v) paraformaldehyde (Sigma) and 2.5% (v/v) glutaraldehyde (Sigma) in phosphate buffered saline (PBS, Sigma). After placing in vacuum for 2 h and overnight incubation, the material was rinsed in PBS (three times, 15 min each). Dehydration (in a graded series of ethanol solutions from 30%, 50%, 70%, 90%, to 99.8% for 20 min) was carried out at room temperature. Ethanol was substituted with 3:1, 1:1, and 1:3 mixtures of EtOH and LR White resin (Sigma) for 2 h and with pure LR White resin overnight. The polymerisation in gelatine capsules was performed at 55 °C for 48 h. Semithin (1 µm) sections were obtained using an ultramicrotome equipped with a glass knife. The sections were mounted on poly-l-lysine coated glass slides (Sigma). Slides prepared for immunochemistry reactions were circled with a liquid blocker PAP Pen (Sigma). Sections adhering to poly-l-lysine slides were washed in PBS twice and treated with 1% bovine serum albumin (BSA) in PBS for 30 min to avoid nonspecific binding of antibodies. Then, the sections were incubated with the primary antibody present at a 1:50 dilution in 0.1% BSA at 4 °C for 24 h and washed four times with PBS. The experiment was conducted using JIM13, LM2, LM14 monoclonal antibodies. Importantly, goat anti-rat IgM conjugated with AlexaFluor 488 was used as a secondary antibody for visualisation of the examined epitopes (cat. no. A21212; Thermo Fisher Scientific, USA). The secondary antibody was diluted 1:200 in 1% BSA at 4 °C and left in darkness. After 24 h, the incubated sections were washed in PBS and deionised water, and finally enclosed in Dako Fluorescent Mounting Medium (Sigma). The observation was carried out using an Olympus BX51 CLSM equipped with corresponding software FluoView v. 5.0. (Olympus Corporation, Tokyo, Japan). All parameters (i.e. laser intensity, gain) were kept constant for all samples. The excitation wavelength for AlexaFluor488 was 490 nm, and the emission was collected at 525 nm. Control reactions were carried out by omitting the primary antibody. Photographs were edited using the CorelDrawX6 graphics program.

### Immunogold-labelling using transmission electron microscopy (TEM)

To analyse the presence of AGPs and other cell wall components at the subcellular level, immunogold-labelling was carried using transmission electron microscopy according to the standard procedure as reported previously^37,38^. Ultrathin sections (70 nm-thick) were cut on an ultramicrotome with a diamond knife and mounted onto formvar-coated nickel square mesh grids (300 MESH). The grids were washed in deionised water and blocked by incubation on a drop of 1% BSA in PBS. Briefly, the grids were incubated at 37 °C for 3 h with primary monoclonal antibodies diluted (1:30) in PBS containing 0.1% BSA. After the reaction with mAb, the sections were washed in 1% BSA in PBS and re-probed with a secondary anti-rat IgG (whole molecule)–gold antibody conjugated with 10 nm colloidal gold nanoparticles (cat. no. G7035, Sigma Aldrich) diluted (1:50) in PBS with addition of 0.1% BSA at 37 °C for 2 h. The grids were post-stained with 1% uranyl acetate for 5 min and Reynold’s reagent for 1 min for better visualisation of the epitopes. The counterstaining was followed by three washes in PBS and five washes in deionised water. Control reactions were carried out by omitting the primary antibody and keeping the rest of the protocol unchanged. The imaging was carried out under a TEM Zeiss EM900 transmission electron microscope (Carl Zeiss AG, Oberkochen, Germany) and equipped with a digital camera with corresponding software ImageSP v. 1.1.2.5.

### Fourier-transform infrared spectroscopy (FT-IR)

A wavelength absorbance spectrum from the samples of AGPs from the green, red, and stored fruits was obtained using a Nicolet 6700 FT-IR spectrometer (Thermo Scientific, Waltham, MA, USA) with Smart *iTR* ATR sampling accessory. The analysis was carried using FT-IR according to the standard procedure as reported previously^39^. Lyophilized samples were applied on ATR and three samples were examined in the same conditions. The spectra were collected over a range of 4000–650 cm^−1^. For each sample, 200 scans were averaged with the resolution of 4 cm^−1^, and then a final average spectrum was calculated. Each sample was performed in triplicate. 10 spectra were made for each repetition, and thus 30 spectra of each sample were averaged. Also, the background was collected after 3 spectra were recorded. Baseline corrections were carried out using Omnic Software (Thermo Scientific). FT-IR spectra were plotted using the OriginPro program (Origin Lab v8.5 Pro, Northampton, USA).

### Atomic force microscopy imaging (AFM)

Atomic force microscope (Multimode 8 with a Nanoscope V controller, Bruker, Billerica, MA, USA) in the semiautomatic high-speed tapping mode was applied to collect AFM height images of AGP samples. Imaging was performed by means of a silicon nitride cantilever with a 2-nm nominal radius of the pyramidal tip, nominal resonance frequency 130 kHz, and a nominal spring constant of 0.4 N/m (ScanAsyst-AIR-HR, Bruker, Billerica, MA, USA). Lyophilised AGP samples were diluted in ultrapure water to the concentration of 0.1 mg ml^−1^ and then subjected to ultrasounds for 10 s and mixed for 2 h. The samples were then deposited on freshly cleaved mica sheets by a spin coater SPIN150i (SPS-EUROPE, Putten, The Netherlands) and dried in a desiccator (relative humidity RH 15%) at 22 °C overnight. At least ten AFM images were collected for each sample. The quantitative analysis of AFM height images was performed using SPIP 6.2.0 software (Image Metrology, Hørsholm, Denmark). First, all images were flattened using third order polynomial fitting to remove the bow effect of the surface. Basic image correction procedures including smoothing and noise reduction by Gaussian (SD 1) and median (3 × 3 weak) filtering was applied.

A “Particle & Pore Analysis” module was used to extract the AGP molecules automatically and calculate their geometric parameters (diameter, area, maximum height, mean height, aspect ratio, compactness, elongation). Description of parameters according to available information in SPIP 6.2.0 software (Image Metrology, Hørsholm, Denmark):

*Aspect ratio* is the aspect ratio defined as Length over Breadth. The aspect ratio will from this definition always be greater than or equal to 1.0. The aspect ratio of both a circle and square is 1.0, whereas other shapes will have a value less than 1.0.

*Compactness* is a measure expressing how compact a feature is. A circle will have a compactness of 1.0, a squares compactness is 1.1284, whereas elongated and irregular shapes result in values less than 1.0.

*Elongation* is a measure indicating how elongated a shape is. A square or circle will return the value zero. As these shapes changes towards a long rectangle or ellipse the returned value converges towards 1.0.

### Statistical analysis

The data were statistically analysed using OriginPro 8.5 software (Origin Lab v 8.5 Pro, Northampton, USA) and STASTICA 18.0 (StatSoft, Inc., 1984–2008). Analysis of variance (one-way ANOVA) followed by post hoc Tukey's honestly significant difference test was used for comparisons of the mean values. For all analyses, the significance level was estimated at *p* = 0.05.
